# Association between lymphocyte and monocyte subsets and cognition in children with HIV

**DOI:** 10.1186/1742-6405-11-7

**Published:** 2014-01-22

**Authors:** Jintanat Ananworanich, Torsak Bunupuradah, Tanakorn Apornpong, Pope Kosalaraksa, Rawiwan Hansudewechakul, Suparat Kanjanavanit, Chaiwat Ngampiyaskul, Jurai Wongsawat, Wicharn Luesomboon, Nicole Ngo-Giang-Huong, Tanyathip Jaimulwong, Stephen J Kerr, Pim Brouwers, William T Shearer, Thanyawee Puthanakit

**Affiliations:** 1 HIV-NAT, The Thai Red Cross AIDS Research Centre, 104 Rajdumri Road, Pathumwan, Bangkok 10330, Thailand; 2 SEARCH, the Thai Red Cross AIDS Research Center, Bangkok, Thailand; 3 Department of Medicine, Faculty of Medicine, Chulalongkorn University, Bangkok, Thailand; 4 Srinagarind Hospital, Khon Kaen University, Khon Kaen, Thailand; 5 Chiangrai Prachanukroh Hospital, Chiang Rai, Thailand; 6 Nakornping Hospital, Chiang Mai, Thailand; 7 Prapokklao Hospital, Chantaburi, Thailand; 8 Bamrasnaradura Infectious Disease Institute, Nonthaburi, Thailand; 9 Queen SavangVadhana Memorial Hospital, Chonburi, Thailand; 10 Institut de Recherchepour le Developpement IRD U174, Program for HIV Prevention and Treatment (PHPT), Chiang Mai, Thailand; 11 Kirby Institute of Infection and Immunity in Society, The University of New South Wales, Sydney, Australia; 12 Division of AIDS Research, National Institute of Mental Health, Bethesda, MD, USA; 13 Baylor College of Medicine, Houston, USA; 14 Department of Pediatrics, Faculty of Medicine, Chulalongkorn University, Bangkok, Thailand

**Keywords:** Children, HIV, Lymphocytes, Monocytes, Cognition

## Abstract

**Background:**

This study assesses the relationships between lymphocyte and monocyte subsets and intelligence quotient (IQ) scores in antiretroviral therapy (ART)-naive, HIV-infected Thai children without advanced HIV disease.

**Findings:**

Sixty-seven ART-naive Thai children with CD4 between 15-24% underwent cognitive testing by Weschler intelligence scale and had 13 cell subsets performed by flow cytometry including naive, memory and activated subsets of CD4+ and CD8+ T cells, activated and perivascular monocytes and B cells. Regression modelling with log_10_ cell count and cell percentage transformation was performed.

Median age (IQR) was 9 (7–10) years, 33% were male, CDC stages N:A:B were 1:67:31%, median CD4% and count (IQR) were 21 (18–24)%, 597 (424–801) cells/mm^3^ and HIV RNA (IQR) was 4.6 (4.1-4.9) log_10_ copies/ml. Most (82%) lived at home, 45% had a biological parent as their primary caregiver, and 26 (49%) had low family income. The mean (SD) scores were 75 (13) for full scale IQ (FIQ), 73 (12) for verbal IQ (VIQ) and 80 (14) for performance IQ (PIQ). Adjusted multivariate regression analysis showed significant negative associations between B cell counts and FIQ, VIQ and PIQ (p < 0.01 for all); similar associations were found for B cell percentages (p < 0.05 for all).

**Conclusions:**

High B cell counts and percentages were strongly associated with poorer FIQ, VIQ and PIQ scores. Prospective, long-term assessment of cell subsets and determination of relevant B cell subpopulations could help further elucidate associations between lymphocyte subsets and neurocognitive development.

## Findings

### Introduction

HIV causes neurocognitive impairment possibly by transmigration through the blood brain barrier of activated T cells and infected monocytes triggering a cascade of inflammatory processes [1]. HIV encephalopathy, a severe form of neurocognitive impairment in children, has become less common since antiretroviral therapy (ART) became widely available. However, chronic stable cognitive and behavioral deficits remain, predicting academic impairments in school [2]. Limited studies in US children have suggested that certain lymphocyte subsets may predict poorer cognition [3,4]. Here we assess the relationships between lymphocyte and monocyte subsets and intelligence quotient (IQ) scores in ART-naive, HIV-infected Thai children without advanced HIV disease.

### Materials and methods

We utilized data from the baseline visit of a 3-year PREDICT immediate vs. deferred ART study (http://www.clinicaltrials.gov/ct/show/NCT00234091) which enrolled ART-naïve children aged 1 to 12 years with CD4 15-24% and no AIDS illnesses. The data included in this analysis were from Thai children at 7 clinical sites who had both flow cytometry and cognitive testing performed before the initiation of ART. The flow cytometry was done according to Pediatric AIDS Clinical Trials Group procedures at US National Institute of Allergy and Infectious Diseases-certified laboratories with rigorous quality assurance programs [5]. The 13 cell subsets included CD45+/3+/19- (Total T cells), CD45+/3+/4+ (Helper T cells), CD4+/45RA+/62 L + (Naive helper T cells), CD3+/4+/45RO + (Memory helper T cells), CD4+/DR+/38+ (Activated helper T cells), CD45+/3+/8+ (Cytotoxic T cells), CD8+/45RA+/62 L + (Naive cytotoxic T cells), CD3+/4-/45RO + (Memory cytotoxic T cells), CD8+/DR+/38+ (Activated cytotoxic T cells), CD45+/3-/19+ (B cells), CD45+/3-/16+/56+ (Natural killer cells or NK cells), CD14+/16+/DR + (Activated monocytes) and CD14+/16+/163+ (Perivascular monocytes believed to be precursors of brain perivascular macrophages) [6]. Cognitive functioning was assessed with Wechsler Preschool and Primary Scale of Intelligence II for those between 2.5 and 6 years or Wechsler Intelligence Scale for Children III for children ≥ 6 years of age. Quality assurance was performed by a US neuropsychologist.

Children’s characteristics were described by median, interquartile range (IQR) or percentages, as appropriate. Each cell subset percentage and count was used as a continuous covariate. Regression modelling with both log_10_ transformed cell count and cell percentage as outcome variables was performed against IQ scores, and adjustment was made for age, gender, HIV RNA, education of caregiver and socioeconomic status. Analyses were performed with Stata 12.1 (Statacorp, College Station, Tx, USA). The level of significance was based on P value of less than 0.05.

The study was approved by the following institutional review boards in Thailand: the Ministry of Public Health, Chulalongkorn University, Khon Kaen University, Bamrasnaradura Infectious Disease Institute, Chiangrai Prachanukroh Hospital and Queen Savang Vadhana Memorial Hospital.

### Results

A total of 67 ART-naive Thai children were included in the analysis, and characteristics are summarized in Table 1. Their median age (IQR) was 9 (7–10) years, 33% were male, Center for Disease Control and Prevention stages N:A:B were 2:67:31%, median CD4% and count (IQR) were 21 (18–24)% and 597(424–801) cells/mm^3^, and median HIV RNA (IQR) was 4.6 (4.1-4.9) log_10_ copies/ml. Most (82%) lived at home, with 45% having a biological parent as their primary caregiver. Twenty-two (33%) had caregivers who had education equal to high school/bachelor, and 26 (39%) had low or very low family income. Median time (IQR) between IQ test and cell subsets was 3 (1–5) months.

**Table 1:** Socio-demographic and disease-related characteristics of children in the study

**Characteristic**	**N (%) or Median (IQR)**
Age	9 (7 – 10) years
Proportion of females	45 (67%)
Thai ethnicity	67 (100%)
Height for age z-score	−1.28 (−1.88, -0.68)
Weight for age z-score	−1.1 (−1.66, -0.57)
Weight for height z-score	−0.3 (−0.92, 0.14)
CDC disease classification	
N	1 (2%)
A	45 (67%)
B	21 (31%)
CD4 percent	21 (18 – 24)%
Log_10_ plasma HIV-RNA (copies/mL)	4.6 (4.1 – 4.9)
***Transmission route***	
Perinatal infection	67 (100%)
Exposure to nevirapine for PMTCT	6 (9%)
***Income***	
Very low	8 (11.9)
Low	18 (26.9)
Average	25 (37.3)
Above average	2 (3.0)
Unknown	14 (20.9)
***Primary caregiver***	
Parent	30 (44.8)
Grandparent	12 (17.9)
Aunt/Uncle	9 (13.4)
Orphanage	11 (16.4)
Step father/mother or foster mother	3 (4.5)
Others	2 (3.0)
***Education of primary caregiver***	
None	9 (13.4)
Elementary	36 (53.7)
High/vocational	15 (22.4)
Bachelor	7 (10.5)

*PMTCT* prevention of mother to child transmission.

The mean (SD) scores were 75 (13) for full scale IQ (FIQ), 73 (12) for verbal IQ (VIQ) and 80 (14) for performance IQ (PIQ). The median (IQR) scores of each cell subset percentages and counts are included in Table 2.

**Table 2:** Multivariate regression analysis of associations between cell subsets and intelligent quotients

		**Multivariate regression analysis**		
		**Coefficient (95% CI), p value**		
**Cellsubsets**	**Median (IQR)**	**Full-scale IQ**	**Verbal IQ**	**Performance IQ**
**Cell counts**				
Total T	2079 (1547,2666)	−9.8 (−42.8, 23.2), p 0.55	−10.1 (−37.7, 17.6), p 0.46	−10.0 (−46.0, 26.0), p 0.57
B	321 (247,481)	**−40.0 (−63.8, -16.2), p 0.002**	**−31.7 (−52.2, -11.3), p 0.004**	**−37.1 (−62.4, -11.9), p 0.005**
Natural killer	297 (152,386)	7.0 (−8.9, 22.8), p 0.38	3.3 (−10.1, 16.7), p 0.62	8.5 (−8.7, 25.6), p 0.32
Helper T	594 (362,795)	−8.5 (−34.7, 17.6), p 0.51	−8.4 (−30.3, 13.5), p 0.44	−8.4 (−36.6, 19.9), p 0.55
Cytotoxic T	1360 (970,1697)	−3.4 (−36.6, 29.8), p 0.84	−6.7 (−34.5, 21.1), p 0.62	−2.1 (−38.4, 34.3), p 0.91
Naïve helper T	314 (168,417)	−6.5 (−21.5, 8.6), p 0.39	−6.4 (−18.9, 6.2), p 0.31	−5.8 (−22.2, 10.6), p 0.47
Naïve cytotoxic T	311 (240,451)	−5.7 (−27.3, 15.8), p 0.59	−3.4 (−21.5, 14.8), p 0.70	−6.9 (−30.6, 16.8), p 0.55
Activated helper T	67 (39,98)	4.4 (−16.5, 25.3), p 0.67	2.9 (−14.7, 20.4), p 0.74	1.9 (−20.0, 23.8), p 0.86
Activated cytotoxic T	584 (460,865)	−7.7 (−38.1, 22.7), p 0.61	−6.6 (−32.2, 18.9), p 0.60	−11.1 (−43.4, 21.2), p 0.49
Memory helper T	228 (168,305)	−2.2 (−30.8, 26.5), p 0.88	−7.0 (−31.0, 16.9), p 0.55	0.7 (−29.4, 30.7), p 0.96
Memory cytotoxic T	683 (543,908)	−27.2 (−57.5, 3.0), p 0.08	**−25.3 (−50.4, -0.3), p 0.048**	−26.0 (−58.3, 6.3), p 0.11
Activated monocytes	58 (33,115)	17.4 (−3.3, 38.1), p 0.10	11.3 (−6.1, 28.7), p 0.19	15.9 (−7.2, 38.9), p 0.17
Perivascular monocytes	31 (12,59)	−4.2 (−15.4, 6.9), p 0.44	−2.9 (−12.1, 6.3), p 0.52	−6.3 (−18.0, 5.4), p 0.28
**Cell percentages**				
CD4/CD8	0.4 (0.4,0.5)	−17.4 (−60.5, 25.6), p 0.41	−11.3 (−47.7, 25.1), p 0.53	−19.7 (−66.6, 27.2), p 0.40
Total T	76.1 (69.5.80.3)	11.1 (−121.0, 143.2), p 0.86	10.2 (−100.8, 121.2), p 0.85	8.0 (−131.3, 147.2), p 0.91
B	12.4 (9.3.15.9)	**−43.5 (−71.8, -15.1), p 0.004**	**−32.2 (−56.9, -7.5), p 0.012**	**−45.5 (−76.5, -14.5), p 0.005**
Natural killer	8.5 (5.8,12.3)	11.9 (−5.6, 29.5), p 0.17	7.4 (−7.5, 22.4), p 0.32	14.3 (−5.0, 33.5), p 0.14
Helper T	20.6 (16.1,22.9)	−8.6 (−64.0, 46.8), p 0.75	−7.3 (−53.8, 39.3), p 0.75	−8.7 (−69.9, 52.6), p 0.77
Cytotoxic T	50.3 (44.5,54)	37.4 (−37.8, 112.5), p 0.32	21.0 (−42.7, 84.7), p 0.51	43.4 (−37.0, 123.8), p 0.28
Naïve helper T	49.5 (42,60.2)	−12.2 (−39.8, 15.4), p 0.37	−12.1 (−35.2, 10.9), p 0.29	−10.4 (−40.8, 20.1), p 0.49
Naïve cytotoxic T	24.1 (18.2,29.4)	−9.9 (−42.5, 22.8), p 0.54	−1.2 (−28.9, 26.4), p 0.93	−12.9 (−47.5, 21.6), p 0.45
Activated helper T	10.9 (8.7-15.4)	8.0 (−10.7, 26.7), p 0.39	6.7 (−9.0, 22.4), p 0.39	6.2 (−14.4, 26.8), p 0.54
Activated cytotoxic T	49.7 (39,59.6)	−10.2 (−54.3, 34.0), p 0.64	−2.0 (−39.2, 35.2), p 0.91	−20.6 (−68.0, 26.7), p 0.38
Memory helper T	10.9 (9.9,13.3)	8.0 (−27.5, 43.4), p 0.65	0.7 (−29.1, 30.6), p 0.96	12.4 (−25.6, 50.4), p 0.51
Memory cytotoxic T	34.2 (29.9,41.4)	−26.2 (−63.3, 10.8), p 0.16	−23.2 (−54.2, 7.9), p 0.14	−26.6 (−67.3, 14.0), p 0.19
Activated monocytes	11.1 (8.1,16.7)	22.0 (−3.9, 47.9), p 0.09	18.0 (−3.3, 39.4), p 0.09	18.1 (−11.1, 47.3), p 0.21
Perivascular monocytes	6.5 (2.1,10.1)	−6.7 (−19.4, 6.1), p 0.29	−3.8 (−14.4, 6.9), p 0.47	−9.5 (−22.8, 3.8), p 0.15

Regression analysis was performed with an outcome variable of log_10_ transformed T cell subset percentages and counts, versus cognitive outcomes adjusted for potential confounders (age, gender, HIV RNA, education of primary caregiver and household income).

*IQ* Intelligence quotient.

Multivariate regression analysis was performed to evaluate associations between cell subsets and IQ scores (Table 2). After adjusting for age, gender, HIV RNA, education of caregiver and income, significant negative associations between B cell counts with FIQ, VIQ and PIQ were observed (p < 0.01 for all). Similar negative associations were found for B cell percentages (p < 0.01 for FIQ and PIQ; p < 0.05 for VIQ). There was a marginal inverse association between memory cytotoxic T cell count and VIQ (p = 0.048). The other cell subsets showed no significant associations with IQ scores.

### Discussion

The present study evaluated the associations between 13 peripheral blood mononuclear cell subsets and IQ scores in ART-naive, HIV-infected Thai children with mild to moderate immune suppression. Higher B cell percentages and counts were strongly associated with poorer FIQ, VIQ and PIQ.

B-cell perturbation in particular over proliferation of aberrant B cell populations and immunoglobulins, and depletion of resting memory B cells, is a marker for HIV-associated immune deficiency. Such aberrations are linked to high HIV viremia and low CD4 nadir [7]. Although no published report exists on the association of B cells with neurodevelopment in HIV-infected children, higher B cell counts have been reported in children with autism and postulated to contribute to autoimmune response and neurodevelopment disorder in that disease [8]. B lymphocytes can enter the brain and they tend to display an activated phenotype. In HIV-infected patients, increased numbers of B cells in autopsied brain parenchyma and perivascular spaces are observed [9]; possibly playing a role in brain inflammation and insult leading to poorer neurocognition. The marginal significant association between higher memory CD8+ T cell counts with poorer VIQ could be a coincidental finding seen in untreated HIV disease. Persistent exposure to HIV viremia can lead to preferential depletion of naive CD8+ T cells and skewed maturation of memory CD8+ T cells [10].

We did not observe a negative association between activated cytotoxic T cells and neurocognition as reported in a US study by Mekmullica et al. In that study, the relative absence of activated cytotoxic T cells, CD8 + HLA-DR + and CD8 + 38+ cells, in the first few months of life was strongly associated with favorable psychomotor outcomes in the first 2 years of life [3]. Activated cytotoxic T cells could migrate into the brain and cause production of pro-inflammatory cytokines and neurotoxins resulting in neuronal dysfunction [1]. However, Kapetanovic S, et al. recently reported higher activated cytotoxic T cell percentage, using the same surface markers, to have a neuroprotective effect in a group of older ART-treated, US children with advanced HIV disease [4]. The discrepancies in associations between T cell subsets and neurocognition in these two latter studies and our own study might partially be accounted for by differences in study population in age and HIV disease severity. Children in the Mekmullica study were newborn at the time of lymphocyte collection, whereas those in the study by Kapetanovic ranged from 1 year to teenagers, and were mostly heavily pretreated with advanced HIV disease. In contrast, our study was conducted in children with less advanced disease than those described by Kapetanovic. Although the Mekmullica study states that B cells were enumerated, the results were not included in the paper which might suggest that no significant association was found, but nevertheless precludes direct comparison with the results in our study.

It must be acknowledged that the peripheral blood B cell compartment contains several distinct B cell types, e.g., immature/transitional (CD10^+^, CD27^-^, activated/mature (CD21^lo^ CD10^-^), and resting memory (CD21^hi^ CD27^+^) [11]. None of these subsets were included in the measurements in this report and further studies will be necessary to determine the neurotoxic B cell subpopulation. The presence of activated monocytes has also been associated with neurocognitive impairment but we did not see this in our study. CD163 is a scavenger receptor expressed on monocytes and macrophages that are thought to be precursors of perivascular macrophages that traffic to the brain [6,12]. They are seen in higher frequencies in HIV-infected subjects compared to healthy controls, and increased frequencies are associated with HIV viremia and low CD4 count [12]. Therefore the weak negative association between these perivascular monocytes and PIQ may be due to untreated HIV disease.

Our study is limited by the small number of children. As previously noted, they were relatively well without advanced HIV disease and were not yet treated with ART. Age, ethnicity and environment can affect both cell subset distribution and neurocognition [2,5]. These factors may limit the generalizability of our data to other populations. We did not have data from age-matched HIV negative Thais to evaluate the relationship between these cell subsets and IQ. Inclusion of such data would also have allowed us to control for non-HIV confounders such as socioeconomic status and caregiver’s education.

Nevertheless, we observed a strong association between higher B cells and poorer FIQ, VIQ and PIQ. Long term study of this PREDICT cohort of children randomized to immediate versus deferred ART initiation may enable us to further elucidate these relationships, and identify cellular subsets which could perhaps predict poor neurodevelopment. This in turn, could help identify children that would benefit from early ART and/or close monitoring and intervention to improve neurodevelopment.

## Competing interests

All authors declare that they have no competing interests.

## Authors’ contributions

JA, PB, WTS, TP conceived and participated in the design and coordination of the study. JA, TB, PK, RH, SK, CN, JW, WL, TP carried out the study. JA, TA and SK analyzed the data and drafted the manuscript with input from all authors. NNGH and TJ coordinated the immunologic testing. All authors read and approved the final manuscript.

## Predict study team

Steering committee: P. Phanuphak, K. Ruxrungtham, M. C. Vun,

V. Saphonn, J. Kaldor, and D.A. Cooper

Endpoint review committee: K. Chokephaibulkit, V. Sirisanthana, P. Suntarattiwong, M. Cotton, C. Giaquinto, R. Lolekha.

National Institutes of Health: L. Fox, A. Ojumu, J. E. Bupp, N. Weatherall, M. Ussery, L.M. Mofenson, P. Brouwers, E. Petrakova.

Advisors: W.T. Shearer, V.G. Valcour, R. Paul, K. Pattanapanyasat, N. Sakulploy, J.M. McNicholl, R. Gelman, K. Rattanadilok, P. Klangsinsirikul.

CIP TH001: HIV Netherlands Australia Thailand (HIV-NAT) Research Collaboration, Thai Red Cross AIDS Research Centre and Chulalongkorn University, Bangkok, Thailand; K. Ruxrungtham, J. Ananworanich, T. Puthanakit, C. Thanee, A. Klinklom, C. Pancharoen, T. Bunupuradah, SJ. Kerr, J. van der Lugt, T. Chuenyam, S. Ubolyam, A. Mahanontharit, T. Suwanlerk, J. Intasan, T. Jupimai, P. Intakan, T. Hirunyanulux, C. Sriheara, A. Uanithirat, P. Boonrak, O. Rit-im, C. Phadungphon, W. Thongsee, O. Chaiya, T. Sattong, K. Nantapisan, A. Piromwong, N. Kuljarusiri, S. Aryukarn, S. Sripanom, N. Naknoi, S. Muangtokit, S. Kumkrung, P. Chaemsai.

CIP TH003: Bamrasnaradura Infectious Disease Institute, Nonthaburi, Thailand; J. Wongsawat, R. Sunthornkachit, V. Moolasart, N. Siripongpreeda, S. Thongyen, P. Chathaisong, V. Prommool, D. Suwannamass, S. Waradejwinyoo, N. Boonyarittipat, T. Chiewcharn, S. Likanonsakul, C. Athichathana, B. Eampokalap, W. Sanchiem.

CIP TH004: Srinagarind Hospital, Khon Kean University, KhonKean, Thailand; P. Kosalaraksa, P. Lumbiganon, C. Engchanil, P. Tharnprisan, C. Sopharak, V. Lulitanond, S. Khahmahpahte, R. Kaewmart, P. Chaimanee, M. Sala, T. Udompanit, R. Wisai,S. Rattanamanee, Y. Chantarasuk, S. Sarvok, Y. Changtrakun,S. Kunhasura, S. Kamollert.

CIP TH005: Queen SavangVadhana Memorial Hospital, Chonburi, Thailand; W. Luesomboon, P. Eiamapichart,T. Jadwattanakul, I. Limpet-ngam, D. Naraporn, P. Mathajittiphun, C. Sirimaskul, W. Klaihong, P. Sittisak, T. Wongwian, K. Charoenthammachoke, P. Yodpo.

CIP TH007: Nakornping Hospital, ChiangMai, Thailand; S. Kanjanavanit, M. Ananthanavanich, P. Sornchai, T. Namwong, D. Chutima, S. Tangmankhongworakun, P. Yingyong, J. Kasinrerk, M. Raksasang, P. Kongdong, S. Khampangkome, S. Thong-Ngao, S. Paengta, K. Junsom, R. Khuankaew, P. Moolsombat, D. Khuttiwung, C. Chanrin.

CIP TH009: Chiangrai Prachanukroh Hospital, ChiangRai, Thailand; R. Hansudewechakul, Y. Jariyapongpaiboon, C. Chanta, A. Khonponoi, C. Yodsuwan, W. Srisuk, P. Ussawawuthipong, Y. Thaweesombat, P. Tongsuk, C. Kumluang, R. Jinasen, N. Maneerat, K. Surapanichadul, P. Donkaew.

CIP TH012: Prapokklao Hospital, Chantaburi, Thailand; C. Ngampiyaskul, N. Srisawat, W. Chamjamrat, S. Wattanayothin, P. Prasertphan, T. Wongcheeree, P. Greetanukroh, C. Imubumroong, P. Teirsonsern.
